# Online reading habits can reveal personality traits: towards detecting psychological microtargeting

**DOI:** 10.1093/pnasnexus/pgad191

**Published:** 2023-06-07

**Authors:** Almog Simchon, Adam Sutton, Matthew Edwards, Stephan Lewandowsky

**Affiliations:** School of Psychological Science, University of Bristol, Bristol BS8 1QU, UK; Department of Computer Science, University of Bristol, Bristol BS8 1QU, UK; Department of Computer Science, University of Bristol, Bristol BS8 1QU, UK; School of Psychological Science, University of Bristol, Bristol BS8 1QU, UK; School of Psychological Science, The University of Western Australia, Perth 6009, Australia; Department of Psychology, The University of Potsdam, Potsdam, Germany

**Keywords:** text modeling, personality, social media, microtargeting

## Abstract

Building on big data from *Reddit*, we generated two computational text models: (i) Predicting the personality of users from the text they have written and (ii) predicting the personality of users based on the text they have consumed. The second model is novel and without precedent in the literature. We recruited active Reddit users (N=1,105) of fiction-writing communities. The participants completed a Big Five personality questionnaire and consented for their Reddit activity to be scraped and used to create a machine learning model. We trained an natural language processing model [Bidirectional Encoder Representations from Transformers (BERT)], predicting personality from produced text (average performance: r=0.33). We then applied this model to a new set of Reddit users (N=10,050), predicted their personality based on their produced text, and trained a second BERT model to predict their predicted-personality scores based on consumed text (average performance: r=0.13). By doing so, we provide the first glimpse into the linguistic markers of personality-congruent consumed content.

**Significance Statement**

In recent years, there has been a growing concern over “psychological microtargeting”, in which psychological features that cannot be directly observed, such as personality characteristics, are inferred from online behavior and personal data, and are used to customize manipulative messages, for example, to provoke political action/inaction or to spread misinformation. Such microtargeting is opaque to online users and although its effects are not fully understood, enough is known to elicit concern. Hence, there is an urgent need to “reverse engineer” microtargeting strategies by uncovering the targeting algorithms in action. This article presents a proof of concept for such algorithmic reverse engineering. We suggest that the detection of personality-congruent language can inform future interventions to alert users when they might be targeted on the basis of their inferred personality.

## Introduction

Social networking websites have the capacity to model online behavior of their users and extract latent psychological representations of such behavior. For example, if an algorithm is provided with 300 Facebook “likes” it can infer a user’s personality with greater accuracy than their own spouse (1). There is a growing concern that these inferred psychological features are used to manipulate users into political action or inaction by delivering persuasive messages that are “psychologically microtargeted”. For example, constructing personality-concordant messages without user awareness or control in an attempt to increase their persuasive impact (2) is arguably a manipulative tactic that harms the democratic process (3, 4).

In this article, we aim to lay the grounds for a tool that would help users understand when they might be manipulated by customized text based on their personality. Building on natural language processing (NLP) models informed by the language users produce and consume online, we can potentially “reverse engineer” the process of psychological microtargeting, thereby boosting individuals’ decision-making autonomy online by alerting them to potentially manipulative content. To our knowledge, we present the first attempt to link attributes of text that people prefer to consume to their underlying personality.

One concerning aspect of psychological microtargeting is that it can exploit individual psychological attributes that cannot be directly observed, such as personality characteristics, by inferring them from online behavior and personal data. Those inferred characteristics are then used to create tailor-made messages. At first glance, this differs little from the long-standing practice of advertisers to segment their audiences according to demographics and coarse indicators of interest—that is why we will not find too many cosmetic ads in motorcycle magazines. However, with the rise of social media, it became apparent that big data enables the construction of a much richer set of features associated with each user’s profile, including covert psychological attributes. For example, Facebook “likes” have been found to be more predictive of users’ personality than most of our real-world social connections (1). Specifically, a machine learning model was trained to predict Big Five personality traits based on users’ likes. Even when the algorithm was trained on only a few dozen likes, it was already a more accurate judge of personality than friends or colleagues; with knowledge of 300 likes the machine outperformed people’s spouses. The success of predictive personality models reveals the capabilities of the platforms and their possible monetization. Facebook registered a psychological microtargeting patent as early as 2014 (5); however, we do not know if it is used or in what settings.

Personality is defined as the long-lasting qualities and behavior that make up an individual’s unique way of responding to life, such as hobbies, motivations, beliefs, self-image, talents, and emotional makeup. These features have long been used to build identity- and values-concordant messages to increase persuasion. The effectiveness of such customization has been examined in several studies in the context of consumer behavior (6, 7). One notable large-scale experiment on Facebook (8) demonstrated that personality-congruent messages increase the likelihood of purchases of cosmetic products (for critical commentaries, see 9, and for a rebuttal by the original authors, see 10).

Another recent finding suggests that personality-congruent messages may capture more attention and increase user engagement but do not show a consistent change in attitudes (11). In political contexts, by contrast, personality-congruent messages have been shown to be more persuasive to online users (2, 12). However, the overall efficacy of such attempts is still debated. While some research has indicated that the concern over political advertising may have been exaggerated (13), recent evidence suggests that, in some contexts, machine learning-driven political microtargeting could potentially outperform other messaging strategies by an average of 70% in shaping public attitudes towards US policy issues (14). Overall, a recent systematic review confirmed that message-tailoring (i.e. aligning messages with characteristics like the personalities of the target audience) is an effective persuasion strategy (r=0.17) (15).

Political microtargeting gives rise to ethical concerns. While it may effectively increase support for causes that are widely seen as meritorious, such as support for climate change action (12) and vaccine uptake (16), these practices may also be enabling duplicity on the part of political actors that could well be viewed as unethical manipulation. For example, microtargeting may be used to discourage individuals from voting in an election or it might be used to incite hatred against outgroups. Therefore, engaging in political microtargeting is ethically subjective and context-dependent, regardless of the specific issue under consideration. While it can be used for informative engagement, as a general practice microtargeting raises concerns about privacy, manipulation, and exploitation of vulnerabilities. Selectively targeted messages, released only to receptive populations and not the public as a whole, could enable actors to undermine democratic processes. Although legally users may have consented to platforms using their data for such practices, it is unlikely that users would willingly donate their online behavior for actions posing a threat to the democratic process (3). Recent research suggests that users in the United Kingdom, United States, and Germany are uniformly reluctant for their online footprints to be used in constructing microtargeted political messages (17).

The ethical question regarding the conceptual and practical differentiation between unethical manipulation and ethical persuasion (18, 19) is beyond the scope of the current article. However, given the prospect of such unethical manipulations, the current work explores possible avenues for mitigation and offers a computational approach for future detection of such attempts.

It has been suggested that an effective method to counteract psychological microtargeting is through “boosting” users’ ability to make informed decisions. Building on inoculation theory (21, 20), Lorenz-Spreen et al. (22) found that a simple intervention, such as providing information about their personality, leads the person to detect microtargeted advertisements with greater accuracy. That is, participants who were given an explanation of the introversion-extraversion dimension of the Big Five together with feedback on their own score, subsequently detected ads that were designed to be particularly persuasive for their personality (introvert or extrovert) with greater accuracy than participants in a control group who received irrelevant information. Although the effectiveness of this intervention attests to the possibility of boosting users’ ability to spot manipulation, it is of limited scalability because it relies on carefully crafted stimuli and requires users to respond to a personality inventory. Both of those limitations can be circumvented through computational modeling of the language of online users and its association with personality.

State-of-the-art NLP models such as Bidirectional Encoder Representations from Transformers (BERT; 23) are pretrained to create context-sensitive vector representations for words (e.g. “a dog’s bark” is different from “the bark from a tree”). When users’ personality scores are regressed onto those vector representations of produced text, the average effect size is r=0.39 (24), demonstrating that knowledge of a user’s produced text permits inferences about their personality.

The current article aims to lay the groundwork to reverse-engineer psychological microtargeting algorithms. Our goal is to detect content that appears to be tailor-made for individuals based on their psychological characteristics without requiring any input from users and without having access to any personal data. Leveraging a deep contextual language model (BERT) (23), we created and fit two models. Model 1 predicts users’ personality traits based on their produced text and is built from a sample of participants who responded to a personality inventory and consented for their text to be analyzed. Model 2 predicts the personality traits of users based on the text they choose to consume and was built without requiring user responses, relying instead on Model 1 to predict their personalities based on produced text.

## Methods

### Overview

We focused on four online communities of fiction-writing on Reddit (WritingPrompts, redditserials, nosleep, and shortstories). Reddit is an online social network of independent communities, each maintaining its own forum where users can post and comment on messages. The fiction-writing communities involve posts that constitute the fiction (e.g. short stories), which are then commented on by users. The users in each community thus both choose to consume text (the fiction) and to produce text (the comments on that fiction). Fiction-writing communities are an excellent target for modeling text because they offer a clear distinction between consumption and commenting. Unlike other subreddits, where the discussion is often a stream of consciousness, in fiction-writing communities, there is a designated, identifiable piece of text that people consume before they comment on it. This makes it easier to study and understand the language that is specifically appealing to different individuals.

**Fig. 1.**
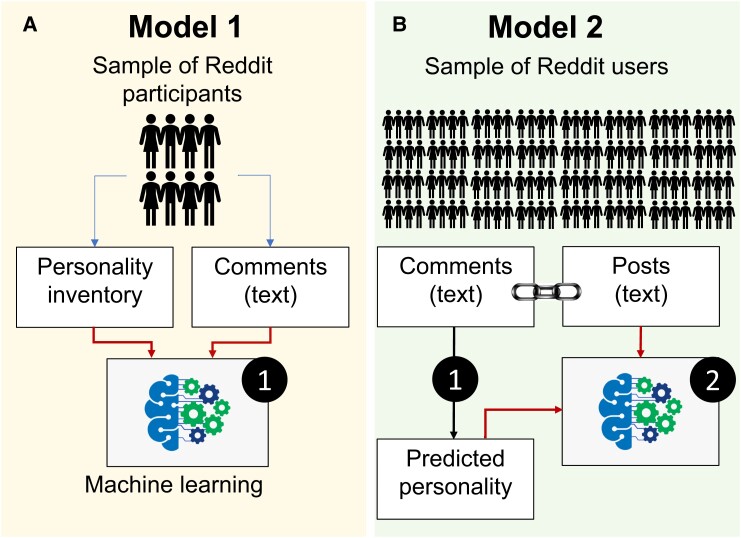
Schematic depiction of Models 1 (A) and 2 (B). Model 1 takes produced text as input and learns to predict people’s personality measured by BFI-2. Model 2, by contrast, takes consumed text as input and learns to predict people’s personality as inferred from Model 1. Model 1 requires participation in the personality inventory whereas Model 2 is built without soliciting responses from any users.

Figure 1 shows the workflow of the current study. First, we invited users to participate in a study to build Model 1. Users responded to a Big Five personality questionnaire (25) and their Reddit comments were scraped to fit a model predicting personality from their authored text (Model 1). Model 1 was then used to predict the personalities of a larger sample of users based on their scraped comments. Those predicted scores, in turn, were used to train Model 2 to link consumed text (i.e. the fiction commented on) to the (predicted) personality. Model 2 thus did not require any user input other than the freely available text they chose to produce in response to a piece of fiction. If successful, Model 2 would enable the detection of consumed text (e.g. in political messages) that matches a user’s personality particularly well, which may signal an attempt at manipulative persuasion.

### Model 1

#### Participants

We invited users who are active contributors to the fiction communities to participate in the study by contacting them via direct messages. We provided details of the study, a unique link to participate, and offered participants the opportunity to enter a raffle of $20 on Amazon as compensation (1 in every 10 participants won a gift card). We sent private messages to 9,475 users, out of which 12% (N=1,105^1^ ) opted in and completed a full personality questionnaire (BFI-2; 25). To maintain user anonymity, we did not collect any demographic information. For more details, see Supplementary Table S1 and Supplementary Fig. S1 in the Supplementary Materials.

#### Data collection

Comments (N=695K) were crawled using the Reddit API and *praw*^2^, a python wrapper used to access the API. All comments users made on all subreddits were crawled and cleaned (i.e. handling emojis, removing capitalization and invalid Unicode words) to provide clear textual representations. The data collected were split into a training set containing 80% of total users surveyed and a test set with the remaining 20% of users.

#### Model structure

We used variations of BERT (23) to fit our predictive models. BERT is a transformer-based machine model with unique architecture: it comprises a deep neural network of 12 layers that was pretrained on large corpora such as Wikipedia and books from various genres. The standard way of using BERT requires “fine-tuning” the pretrained model on a specific task like classification or regression, such that the fully connected network is involved in prediction based on supervised learning.

In the current model, we utilized a variant that leverages the BERT architecture but with fewer layers and parameters (26). We fine-tuned five different models to predict the score of each personality factor by linking comments of fiction consumers to their personality inventory (one model for each factor). Each user contributed up to 100 comments as input into the model. Predictions were generated for every comment and then averaged along each personality dimension. The final output was a 5D vector with predicted personality for each user.

### Model 2

#### Data collection

We identified users with a history of commenting in our four fiction communities. We then collected the pieces of fiction these users had commented on (i.e. their consumed text), and their comments from their general Reddit activity (produced text, extending beyond the fiction communities if users commented more broadly). This was done via the Reddit API (see Model 1 Data Collection) and through querying a large dataset of precollected Reddit activity (27) that is freely available on Big-Query,^3^ a Google-based data warehouse that allows for large-scale data wrangling. The total dataset contained 3.5M comments written by 25,000 users who consumed 28,000 pieces of fiction.

#### Model structure

Our modeling approach differed from Model 1. Instead of fine-tuning a fully connected network, we employed a downstream modeling technique, following a recent precedent of psychological research in NLP (28). This approach is less computationally demanding than fitting a fully connected network and was found to outperform other systems in preliminary tests. The standard BERT architecture comprises 12 embedding layers. Recent findings suggest that BERT layers follow a hierarchical order, with word-level information mostly encoded in the bottom layers, syntactic information mostly encoded in the middle layers, and more complex and contextual information mostly encoded in the top layers (29). Leveraging BERT, we extracted the 11th and 12th layers of the averaged embedding for each text. This resulted in a vector of 1,536 latent features of each text which were then used in an Elastic Net regression to predict the predicted-personality scores.

To avoid an hierarchical structure or giving undue weight to “supercommenters” (highly engaged users), we created a dataset that mapped a single piece of fiction to a single user. This was achieved by randomly sampling one piece of consumed text per user. In the event of duplicated text, we randomly sampled one user from the reduced list of consumed fiction texts. In addition, we refined our decision rule regarding mapping pieces of fiction to users, such that only pieces of fiction that received a positive comment from the user were sampled (we applied sentiment analysis on each comment using the “sentimentr” R package (30)). This decision rule was developed with the idea that we should only include material that users find interesting or enjoyable.

As in Model 1, we fitted five different models to the five personality dimensions. The analysis was carried out with the R packages “text” (31) and “caret” (32).

## Results

### Model 1

#### Model performance

The predictive performance of the model was assessed through one hold-out validation: a random sample of 80% of the data (i.e. 80% of the users) was used as a training set and 20% as a test set. Table 1 shows the predictive performance for each personality dimension on the test set. Overall, we find the model to predict personality quite well (average performance r=0.33), which is within the expected range according to a recent review of state-of-the-art text-based prediction of personality (24). See also Supplementary Fig. S2.

**Table 1.** Match between the predicted personality from model 1 and the “ground truth” personality measure based on the 20% test set. Performance is measured by Pearson’s correlation coefficient and root mean square error (RMSE). RMSE CIs were bootstrapped using 1000 resamples.

Personality dimension	Pearson’s *r* [95% CI]		RMSE [95% CI]	
	Mean	[95% CI]	Mean	[95% CI]
Extraversion	0.26	[0.13, 0.38]	9.59	[6.38, 7.88]
Agreeableness	0.35	[0.23, 0.46]	7.14	[8.38, 10.00]
Conscientiousness	0.37	[0.25, 0.28]	9.24	[8.81, 10.33]
Neuroticism	0.28	[0.15, 0.40]	10.53	[9.65, 11.30]
Openness to experience	0.39	[0.28, 0.50]	7.29	[6.37, 8.20]

#### Visualizing the model

**Fig. 2.**
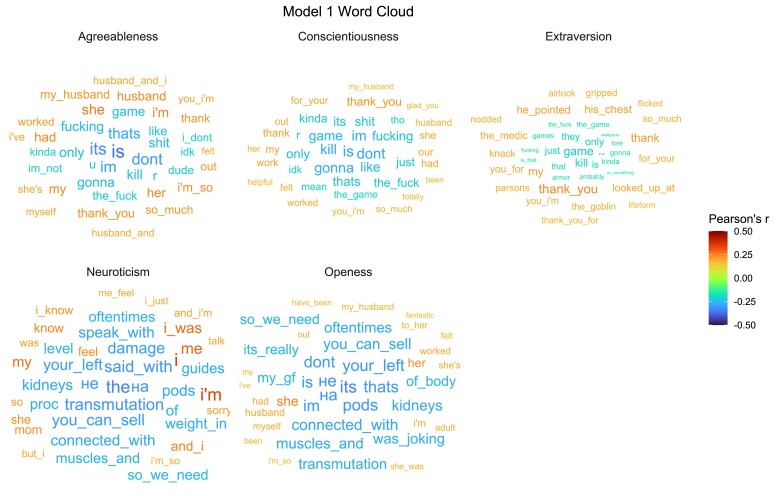
Word cloud of Model 1 showing the top 20 positive and top 20 negative features that best predict each personality dimension. The size of the word represents the effect size (absolute value of *r*) and the color denotes direction (positive or negative).

One limitation of using advanced neural networks in text modeling is the lack of model transparency. In order to get a better understanding of what particular linguistic features are associated with the different personality types, we conducted an N-gram analysis (*N* = 1–3) to find the words, word pairs, and word triplets that best correlated with the model’s predicted personality. The word clouds in Fig. 2 provide a visual description of these relationships. We created a document-feature matrix of the N-grams and correlated the predicted personality score with each feature (N=97,979). The word clouds present the top 20 positive (i.e. r>0) and top 20 negative (r<0) features that exceeded the significance threshold. The size of the word represents the effect size (absolute value of *r*) and the color denotes direction (positive or negative). For other visualizations see Supplementary Figs. S3 and S4 and the online OSF repository.^4^

While some words in the word clouds are easier to interpret (e.g. positive association with gratitude in agreeableness; negative association with violence in conscientiousness), many seem puzzling at first glance, especially pronouns and function words. However, style words (or “stealth words”) have been shown to be predictive in the context of mental health, gender differences, emotional states, and personality (34, 33, 35). Our modeling shows that neuroticism is the only personality factor where singular first-person pronouns (I-words) have a positive loading. This is in line with prior research showing that neuroticism is highly correlated with anxiety and depression (36), psychopathologies that manifest in greater use of singular first-person pronouns (38, 37, 33).

### Model 2

#### Model performance

The performance of Model 2 was assessed via 5-fold cross-validation (i.e. a machine learning technique in which the data is split into five equal parts, and the model is trained and evaluated five times, with each part being used once as the validation set and the rest as the training set). The final dataset comprised 10,050 unique users and a corresponding set of 10,050 unique pieces of fiction. We fitted an Elastic Net regression using a search grid of alpha [0,1] and lambda that ranged from 10${}^{-16}$ to 1 with increments of 0.11. The average performance of the cross-validation was *r* = 0.132, providing a small but reliable link between the text people consume and their predicted personality traits.

Upon choosing the final model, we applied Model 2 to our original Model 1 sample: 1,105 users for which we have personality scores measured by the BFI-2 (25). The final validation set included 939 users for which we could identify both personality scores and consumed text. On average, We find a positive association between the Model-2-predicted personality based on the text users have read, and their ground truth personality measured by the personality inventory (r=0.051). However, only two personality dimensions are significantly correlated with the ground truth estimates (neuroticism and openness); see full results in Table 2 and Supplementary Figs. S5 and S6. While these effect sizes are clearly small, as we argue in the Discussion, even small effect sizes may become consequential at scale (39).

**Table 2.** Match between the predicted scores of predicted personality and ground truth personality. Values in brackets denote 95% CIs. 10,050 observations were included in the 5-fold cross-validation. “Ground truth” refers to our seed sample (based on 939 unique users). Performance is measured by Pearson’s correlation coefficient and root-mean-square error (RMSE). RMSE CIs were bootstrapped using 1000 re-samples.

Measure	Personality dimension	5-fold cross-validation [95% CI]		Ground truth [95% CI]	
Pearson’s *r*	Extraversion	0.08	[0.06, 0.10]	− 0.00	[−0.06, 0.06]
	Agreeableness	0.12	[0.10, 0.14]	0.05	[−0.01, 0.12]
	Conscientiousness	0.12	[0.10, 0.14]	− 0.01	[−0.07, 0.05]
	Neuroticism	0.19	[0.17, 0.20]	0.11	0.05, 0.18]
	Openness to experience	0.15	[0.13, 0.17]	0.10	[0.04, 0.17]
RMSE	Extraversion	9.52	[6.78, 13.25]	11.25	[10.86, 12.78]
	Agreeableness	7.80	[6.93, 9.97]	10.12	[9.72, 11.87]
	Conscientiousness	8.91	[7.31, 10.71]	11.57	[11.1, 12.27]
	Neuroticism	7.61	[6.71, 9.72]	12.34	[11.84, 13.28]
	Openness to experience	6.97	[6.93, 7.99]	15.70	[14.03, 17.65]

#### Visualizing the model

**Fig. 3.**
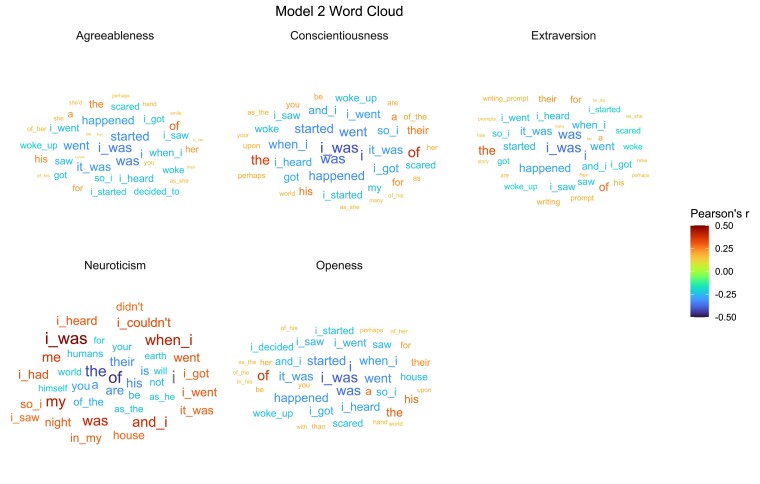
Word cloud of Model 2 showing the top 20 positive and top 20 negative features that best predict each personality dimension. The size of the word represents the effect size (absolute value of *r*) and the color denotes direction (positive or negative).

Following the visualization pipeline of Model 1, we used another N-gram analysis to find the words, word pairs, and word triplets that best correlated with the model’s predicted personality. The word clouds in Fig. 3 provide a visual description of these relationships. We created a document-feature matrix of the N-grams and correlated the personality score with each feature (N=150,772). The word clouds present the top 20 positive and top 20 negative features that exceeded the significance threshold. The size of the word represents the effect size (absolute value of *r*) and the color denotes direction (positive or negative). For other visualizations and analyses see Supplementary Materials, Supplementary Figs. S7–S9, Supplementary Table S2, and the online OSF repository.

Upon inspection, the elements of language that best predict Model 2 consists of function words, pronouns, and features that reflect linguistic style. One possible interpretation is that individuals who exhibit a high score on the neuroticism scale are more likely to read texts that contain self-centered language, which is reflective of their own linguistic traits.

#### Human validation of model 2

To assess the validity and generalizability of Model 2, we collected over 1,500 political ads from Facebook’s Ad Library that targeted UK users (ads were posted between December 2019 and December 2021). We sought to validate Model 2 by focusing on the Openness dimension, which was not only predicted well but is also easy to explain to nonexpert human judges. We applied Model 2 to the ads’ text, predicting the text’s appeal on the openness dimension. Building on the model predictions, we selected 20 ads from the upper decile and 20 ads from lower decile of the predicted openness score and requested participants (*N* = 50; Prolific UK) to provide judgments of the perceived openness of the ad. Our findings corroborated the Model’s prediction, as ads in the top decile of predicted openness received significantly higher openness judgments (b=0.7, p<0.001); see Supplementary Fig. S10. More details can be found in the Supplementary Materials. The results confirm that even though Model 2’s performance appears modest when assessed by correlation coefficient, it can classify real-life political ads in a manner that is consonant with human judgment.

## Discussion

We identified linguistic patterns that correlate with the text people of certain personality characteristics produce (write) and consume (read). We fitted two machine learning models: Model 1 predicted personality traits based on the text people produced and reached state-of-the-art predictive performance (r=0.33). The second model (Model 2) predicted the personality traits of online users based on the text they read. To permit upscaling, and to mimic a real-life situation in which we have no access to the ground truth of users’ personality, Model 2 used the predictions from Model 1 as a target for learning.

Given the novelty of the task, there is no benchmark for its predictive ability. However, the application of Model 2 to the “ground truth” data showed that despite being trained on the predictions of another model, it significantly predicted neuroticism and openness to experience. In addition, this approach has outperformed an attempt to model text consumption with “ground truth” data (Model 3), likely due to the striking differences in available training data (see Supplementary Table S3 and more details in Supplementary Material). Taken together, while Model 2’s cross-validated predictive performance of r=0.13 may be considered low in other tasks, human validation showed it to be generalizable (viz. to political content), and to be in line with human judgments. We believe this is an encouraging result for future work on algorithmic reverse engineering. In the current work, we focused on textual input; however, the digital traces social networks possess are far richer than plain text (e.g. dwell time, passive consumption, engagement, etc.). Leveraging these in future research could shed more light on the nuanced strategies of psychological microtargeting. However, it is crucial to acknowledge that several successful models that utilized metrics like Facebook likes (e.g. (1)) were platform-specific. In contrast, our approach has demonstrated generalizability across platforms (reddit vs. Facebook) and types of content (fiction vs. political ads). This broader applicability enhances the model’s versatility and usefulness across diverse online environments.

One striking difference between the models lies in the linguistic features they load onto. Model 1 (produced text) seemed to be much more oriented toward content (e.g. language that describes interest in video games and family life; use of profanity and appreciative words), whereas Model 2 (consumed text) gravitated towards style (e.g. pronouns, function words, etc.). Interestingly, as revealed by Model 2, people who score high on the neuroticism scale are more prone to consume text that reflects self-centered talk, which is a feature of their own linguistic characteristics. This is just one illustration of how Model 2 captures stylistic elements that appear to span numerous domains and are content-independent. The model’s generality is thus one of its advantages despite the modest effect size.

**Fig. 4.**
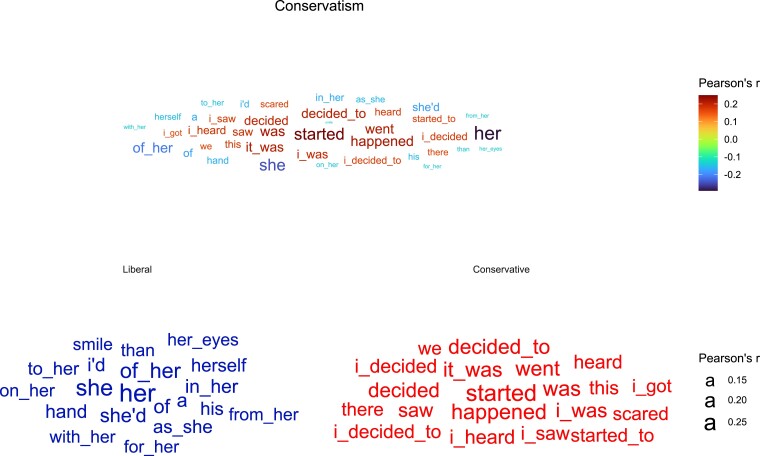
Word clouds of Model 2 predictions of political attitudes. The upper panel represents the top 20 positive and top 20 negative features that best predict political personalities derived from (40). The size of the word represents the effect size (absolute value of *r*), and the color denotes direction (positive for conservatives or negative for liberals). The lower panels show the features separately for liberal personalities and conservative personality. The size of the word represents the effect size.

One implication of Model 2 is the possibility to derive the linguistic features of other constructs of interest that correlate with personality. For example, prior research shows that Big Five personality traits can be used to predict political attitudes (40, 41). To explore the implications of this association, we constructed a political attitude score, based on a weighted average of predicted personality (weights were derived from the correlations reported in 40; for more information see Supplementary Material). Following the same procedure of visualizing Models 1 and 2, we created a document-feature matrix of the N-grams and correlated the predicted political attitude score with each feature. Figure 4 shows the linguistic features that best represent consumed text of “liberal personalities” vs. “conservative personalities”. According to this analysis, liberal personalities show greater use of feminine language (e.g. “her” “she”) whereas conservative personalities demonstrate a preference to declarative, authoritative language (e.g. “decided”, “started”, etc.). Even though this particular analysis should be interpreted with caution because we inferred political attitudes rather than measuring them directly, it exemplifies what this line of research may offer in the future. There are multiple avenues to extend the approach to other domains such as coping with uncertainty (42), basic human values (43), paranormal and superstitious beliefs (44), and so on. The clear differentiation between the two clusters in Fig. 4 also helps allay fears about the small correlations observed for Model 2: notwithstanding the seemingly small effect sizes, the model produced quite different words for the two personality profiles.

A further common aspect of both models is the inverse relationship between the features that best predict neuroticism vs. the rest of the Big Five factors. The notion that neuroticism is distinct from the rest of the factors is not unique to our analysis. Empirical work suggests that a General Factor of Personality (GFP) is at the highest level in a hierarchy of personality structures (45, 46). Importantly, the GFP contrasts neuroticism with conscientiousness, agreeableness, extraversion, and openness, suggesting a single continuum of a general, noncognitive dimension that reflects stability and plasticity, respectively. Our predictive language models of personality appear to reflect the same type of dimensionality reduction, despite having been trained on a different personality factor each time (i.e. different models for each personality dimension).

Taken together, our work supports the potential efficacy of microtargeting: it suggests that people choose to read text that is consonant with their personality (at least some of the time), and this may make certain messages more palatable to an individual than others. A major concern remains that political operatives or bad-faith actors could exploit this to undermine the democratic process. However, while this may be a valid concern, the extent of such practices remains unclear. With the increasing influence of social media on our political landscape, it is crucial that the algorithms that gather those personal data and permit targeting be subjected to auditing. Absent such audits, algorithmic reverse engineering (such as we present here) is the only potential tool the scientific community has to alert people to opaque tactics such as microtargeting.

## Conclusion

The current article lays the groundwork for future interventions focused on psychological microtargeting. Based on the linguistic features that correlate with personality-congruent messages, future work could build on these findings in developing interventions that could boost people’s decision-making process in online environments. For example, a future browser extension equipped with the present models could gradually learn the personality makeup of users, and alert them when they encounter political ads that seem to be microtargeted at them. This would potentially enhance the user’s autonomy and sense of agency in response to such manipulation attempts.

## Supplementary Material

pgad191_Supplementary_Data

## Data Availability

All shareable data for the study are publicly accessible at https://osf.io/432tb, so are the code and the models. Sensitive data have not been made available on a permanent third-party archive because our institutional review board ruled that we could not post the data. The study herein was not preregistered due to its exploratory nature.

## Footnotes

1 20 users were recruited via other methods on Reddit such as Reddit Ads.

2
https://praw.readthedocs.io/en/stable/

3
https://cloud.google.com/bigquery

4
https://osf.io/432tb
