# Correction to: Online reading habits can reveal personality traits: towards detecting psychological microtargeting

**DOI:** 10.1093/pnasnexus/pgad380

**Published:** 2023-11-15

This is a correction to: Almog Simchon, Adam Sutton, Matthew Edwards, Stephan Lewandowsky, Online reading habits can reveal personality traits: towards detecting psychological microtargeting, *PNAS Nexus*, Volume 2, Issue 6, June 2023, pgad191, https://doi.org/10.1093/pnasnexus/pgad191

During a retroactive audit conducted by *PNAS Nexus*, it was discovered that this paper was missing a statement acknowledging compliance with the *PNAS Nexus* Human and Animal Participants and Clinical Trials policy:

The first part of the study, predicting personality from produced text (Model 1), was fully reviewed by the School of Psychological Science Research Ethics Committee at the University of Bristol, ethics approval #0242. The second part, predicting personality from consumed text (Model 2), did not involve any behavioral data and relied solely on publicly available data sourced from Reddit and was thus exempt from ethics review at the University of Bristol. The third part, behavioral validation of the Model 2's performance, was fully reviewed by the School of Psychological Science Research Ethics Committee at the University of Bristol, ethics approval #12318. All participants have signed informed consent.

This error has been corrected in the original article.

