# Supplementary material for: Online reading habits can reveal personality traits: towards detecting psychological microtargeting
Source: PNAS Nexus. 2023 Jun 7;2(6):pgad191. doi: 10.1093/pnasnexus/pgad191 (PMC10276193; doi:10.1093/pnasnexus/pgad191)
Supplement: pgad191_Supplementary_Data [file pgad191_supplementary_data.pdf]

Supplementary Material of Online reading habits can reveal personality traits: towards detecting psychological microtargeting

Almog Simchon[1], Adam Sutton[1], Matthew Edwards[1], and Stephan Lewandowsky [1,2,3]

[1]University of Bristol

[2]University of Western Australia

[3]University of Potsdam

Supplementary Material of Online reading habits can reveal personality traits: towards detecting psychological microtargeting

Extended Visualizations

While word clouds are a great tool for data visualization, other ways are emerging to support visualizing textual features. One of the ways is ScatterText (Kessler, 2017). In Figure S3 we present an analysis of the top 1,000 predictive features of Model 1 and their prevalence in the corpus. In Figure S7 we present the same analysis for Model 2. Please see the online OSF repository for an interactive version.

The N-gram analysis shown in the word clouds and ScatterText plots provide insight into the model in a bottom-up fashion. Another way to do this is using the Linguistic Inquiry and Word Count (LIWC; Pennebaker, Boyd, Jordan, & Blackburn, 2015), an extensively validated dictionary of several dimensions of textual content, to identify the linguistic and psychological categories that best explain the model's prediction. Figures S4 and S8 show the zero-order correlations of each LIWC category and the corresponding personality score by Model 1 and Model 2, respectively. For Model 1, LIWC scores were obtained for the comments each user wrote and for Model 2, on the fiction they read. In line with the N-gram analysis, the correlational structure reveals a pattern of neuroticism vs. all-other Big Five personality traits. In addition, it also sheds light on the difference between the two models: Model 2 exhibits stronger effects for function words and pronouns, which again supports the interpretation that Model 2 has stronger gravitation toward style than Model 1.

Model 3: Predicting ground-truth personality from consumed text

Model 3 is a model created via an alternative training process, namely an attention mechanism for each piece of consumed text (Lynn, Balasubramanian, & Schwartz, 2020). This gives a weight for how important each text a user has consumed to the final personality prediction. The underlying assumption is that some consumed texts are more informative to the personality prediction than others, which can be considered by applying a hierarchal neural network. This attempt is intended to explore if it is possible to model personality based on consumed text if we remove the noise introduced from using labels generated by Model 1. The difference from Model 2 is that Model 3 was trained entirely within the seed datasets—that is, the data from the participants sampled for Model 1—rather than the larger soft-labeled corpus (i.e., Model 2 data; Model 1's predictions which were used as labels for Model 2). This results in fewer inventories to predict and fewer textual sources. There are 950 participants sampled in this experiment who have commented on a total of 4,450 pieces of fiction. Despite the fact that this model was trained on ground truth data, and applied state-of-the-art modeling, it did not perform well. See results in Table S3.

Human validation of Model 2

Model 2 was entirely data-driven, trained to predict text that appeals to certain personality traits. To assess the validity and generalizability of Model 2, we conducted a study using human judgments on the model's predictions. Since the main driver of our investigation revolves around using microtargeting in political messages, we applied the model to political ads that were posted on Facebook in recent years. We hypothesized that participants would judge the ads as appealing to the personality type in line with Model 2's predictions.

Method

Participants

We recruited 50 UK-based participants through Prolific. Participants (36 women; 14 men) were invited to participate in a 10-minute study, for which they were compensated with £1.50. Participants' age ranged from 21 to 79 ($M = 43.20$; $SD = 15.01$)

Materials

We collected ads from the Facebook Ad Library using their API. We collected political ads that targeted a UK audience between December 2019 and December 2021. We queried the API using specific keywords or specific political pages. The keywords were: *Boris, Immigration, Refugees, Brexit, Government, Minister, MP, Patel, Policy, Tax, Tories, Vote, Parliament, Corbyn, Starmer,* and *Dominic Raab.* The pages we queried were: *Alba Party, Alliance Party of Northern Ireland, Conservatives, Democratic Unionist Party, Green Party of England and Wales, The Labour Party, Liberal Democrats, Plaid Cymru, The Scottish Government, Scottish National Party (SNP), UK Government, The Telegraph, The Times*, and *The Sunday Times.*

Queries using the above search terms and page ids resulted in a total of 7,457 unique ads. We filtered low-spending ads and retained ads that spent at least £100. The final dataset contained 1552 ads. We then applied Model 2 on the entire dataset and

selected 20 ads from the top decile of the predicted openness and 20 ads from the bottom decile of predicted openness as stimuli. We focused on openness because it is one of the two best-predicted personality dimensions, and it is easy to understand and explain, and also non-prejudicial (unlike, e.g., neuroticism).

Procedure

Data was collected through Qualtrics. Participants were provided with the following definition of Openness to Experience and were requested to rate the 40 ads on a 1-6 Likert scale ranging from Strongly Closed to Experience to Strongly Open to Experience:

> One way in which people differ from each other is through their personalities. The Big-Five Personality Model identifies five personality traits on which each individual varies. One of the traits is Openness to Experience.
>
> Openness to experience has both motivational and structural components. People high in openness are motivated to seek new experiences and to engage in self-examination. Closed people by contrast are more comfortable with familiar and traditional experiences.
>
> In the following task you will be presented with a series of political advertisements that have been published in the UK over the last 5 years. For each advertisement, you will be asked to identify to what extent each advertisement is oriented towards people on the Openness to experience scale, ranging from Very Closed (lowest) to Very Open (highest).

The order of the ads was randomized between participants.

Results

We fit a linear mixed model predicting the participants' rating of the ad using Model 2's classification of the ad as fixed factor [top 10% (high on Openness) or bottom 10% (low on Openness)]. We included two random intercepts, one by participant and one by ad. In line with Model 2's predictions, results show a significant effect of ad

classification. The effect of openness classification is statistically significant and positive ($beta = 0.72$, 95% CI [0.43, 1.00], $t(1995) = 4.97$, $p < .001$; $Std.beta = 0.49$, 95% CI [0.30, 0.69]). See ratings in Figure S10.

Predicting political attitudes

To illustrate the potential use of Model 2, we built on the predictive abilities of the Big Five and the association between personality and political attitudes. For each user, we constructed a "political attitude" score, calculated as a weighted average of their personality, predicted by Model 2. The weights of the average were taken from the correlation coefficients reported in (Gerber, Huber, Doherty, & Dowling, 2011). We then followed the same visualization pipeline of Model 1 and Model 2, and used an N-gram analysis ($N = 1 - 3$) to find the words, word pairs, and word triplets that best correlated with the political attitude score, derived from Model 2's predicted personality.

Subreddits by personality

One way to explore the predictive validity of Model 2 is to observe what are the popular subreddits of users based on their predicted personality of consumed text. For every user in Model 2, we extracted the list of subreddits they commented on. Having collected all subreddits, we then created an average “personality rating” for each subreddit. Those averages were converted to z-scores to allow direct comparison between personality dimensions. We found that subreddits like r/science, r/books, and r/writing are popular among users who are high on openness. In contrast, more niche, internet-culture subreddits gain popularity among people who are high on neuroticism (see Figure S9). The 20 most influential subreddits by personality dimension are shown in Table S2.

References

Gerber, A. S., Huber, G. A., Doherty, D., & Dowling, C. M. (2011, June). The big five personality traits in the political arena. *Annu. Rev. Polit. Sci.*, *14*(1), 265–287.

Kessler, J. S. (2017). Scattertext: a browser-based tool for visualizing how corpora differ.

Lynn, V., Balasubramanian, N., & Schwartz, H. A. (2020). Hierarchical modeling for user personality prediction: The role of message-level attention. In *Proceedings of the 58th annual meeting of the association for computational linguistics* (pp. 5306–5316).

Pennebaker, J. W., Boyd, R. L., Jordan, K., & Blackburn, K. (2015). The Development and Psychometric Properties of LIWC2015.

Soto, C. J., & John, O. P. (2017, July). The next big five inventory (BFI-2): Developing and assessing a hierarchical model with 15 facets to enhance bandwidth, fidelity, and predictive power. *J. Pers. Soc. Psychol.*, *113*(1), 117–143.

Table S1

Cronbach's alpha of BFI-2. N = 1,105. To assess the reliability of the BFI-2 (Soto & John, 2017) in our sample, we computed Cronbach's alpha of each personality dimension. All personalty factors were highly reliable.

Personality Dimension	Cronbachs's *alpha* [95% CI]
Extraversion	0.86 [0.85, 0.87]
Agreeableness	0.81 [0.80, 0.83]
Conscientiousness	0.87 [0.86, 0.88]
Neuroticism	0.90 [0.89, 0.91]
Openness to Experience	0.80 [0.79, 0.82]

Table S2

Table of the 20 most and least popular subreddits by personality dimension (based on 11,820 users with Model 2 predictions). The scores are the predicted Z-score of Model 2's prediction (numbers in parentheses denote rank by absolute Z-score). N denotes the number of users who contributed to the score.

	Subreddit	N	Neuroticism	Extraversion	Conscientiousness	Openness	Agreeableness
1	writing	603	-4.98 (1)	4.81 (1)	4.19 (1)	3.8 (1)	3.09 (1)
2	books	613	-1.57 (8)	2.11 (2)	2.5 (2)	2.49 (2)	2.51 (2)
3	politics	780	-2.35 (2)	1.29 (18)	1.66 (7)	1.55 (10)	
4	science	468	-1.41 (12)	1.25 (20)	1.55 (10)	1.77 (7)	2.17 (3)
5	PewdiepieSubmissions	679	1.84 (4)	-1.9 (3)	-2.16 (3)	-1.93 (4)	-1.99 (6)
6	worldnews	1175	-2.16 (3)	1.49 (10)	1.52 (11)	1.49 (12)	1.5 (13)
7	dankmemes	1366		-1.84 (6)	-1.92 (4)	-1.99 (3)	-2.11 (4)
8	TwoXChromosomes	558		1.35 (15)	1.4 (15)	1.7 (9)	2.02 (5)
9	blursedimages	392	1.78 (5)	-1.88 (4)	-1.49 (12)	-1.32 (19)	-1.38 (18)
10	AskOuija	886	1.42 (11)	-1.85 (5)	-1.87 (5)	-1.83 (5)	-1.83 (7)
11	me_irl	593	1.6 (7)	-1.47 (11)	-1.59 (9)	-1.78 (6)	-1.51 (12)
12	Futurology	422	-1.75 (6)	1.77 (7)	1.43 (14)	1.41 (17)	
13	imsorryjon	470		-1.74 (8)	-1.33 (18)		-1.44 (16)
14	entitledparents	860	1.25 (17)	-1.3 (17)	-1.67 (6)	-1.72 (8)	-1.67 (8)
15	DnD	448	-1.45 (9)	1.37 (14)	1.63 (8)	1.53 (11)	1.52 (11)
16	therewasanattempt	436		-1.28 (19)	-1.38 (16)	-1.45 (15)	-1.6 (9)
17	cursedcomments	736	1.42 (10)	-1.58 (9)			
18	facepalm	592				-1.36 (18)	-1.56 (10)
19	nottheonion	596		1.34 (16)			1.5 (14)
20	television	387	-1.38 (13)	1.44 (12)	1.48 (13)	1.47 (13)	1.47 (15)
21	im14andthisisdeep	400				-1.46 (14)	-1.38 (17)
22	teenagers	1375	1.23 (19)		-1.37 (17)	-1.44 (16)	
23	thatHappened	446		-1.41 (13)			
24	niceguys	443	1.36 (14)				
25	Cringetopia	426				-1.31 (20)	-1.35 (19)
26	RoastMe	1045			-1.22 (19)		-1.35 (20)
27	Unexpected	509	-1.31 (15)				
28	HistoryMemes	560	-1.27 (16)				
29	marvelstudios	465	-1.25 (18)				
30	memes	1909			-1.22 (20)		
31	ChoosingBeggars	849	1.19 (20)				

Table S3

Performance table of the predicted personality from Model 3 and the "ground-truth" personality measure based on the 5-fold cross-validation. Performance is measured by Pearson's correlation coefficient and root-mean-square error (RMSE). RMSE CIs were bootstrapped using 1000 re-samples.

Personality Dimension	Pearson's r [95% CI]		RMSE [95% CI]	
	Mean	[95% CI]	Mean	[95% CI]
Extraversion	0.06	[0.00, 0.13]	11.26	[10.82, 11.69]
Agreeableness	0.05	[-0.01, 0.12]	8.99	[8.50, 9.46]
Conscientiousness	0.03	[-0.03, 0.10]	10.81	[10.39, 11.20]
Neuroticism	-0.01	[-0.07, 0.05]	12.30	[11.76, 12.84]
Openness to Experience	0.05	[-0.20, 0.12]	9.39	[8.91, 9.90]

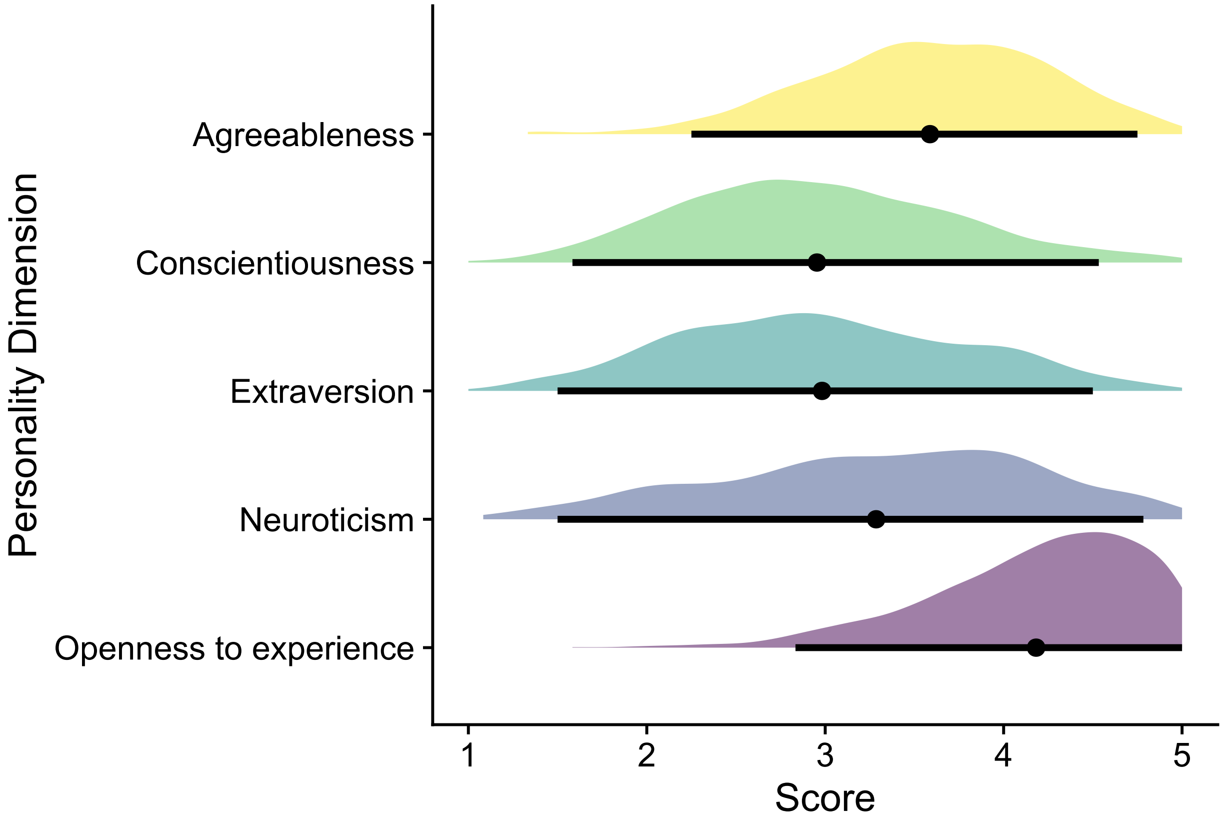

Figure S1

Distributions the personality dimensions among our seed sample (Model 1; N = 1,105). The point estimate corresponds to the mean; intervals denote the 2.5th and 97.5th percentiles.

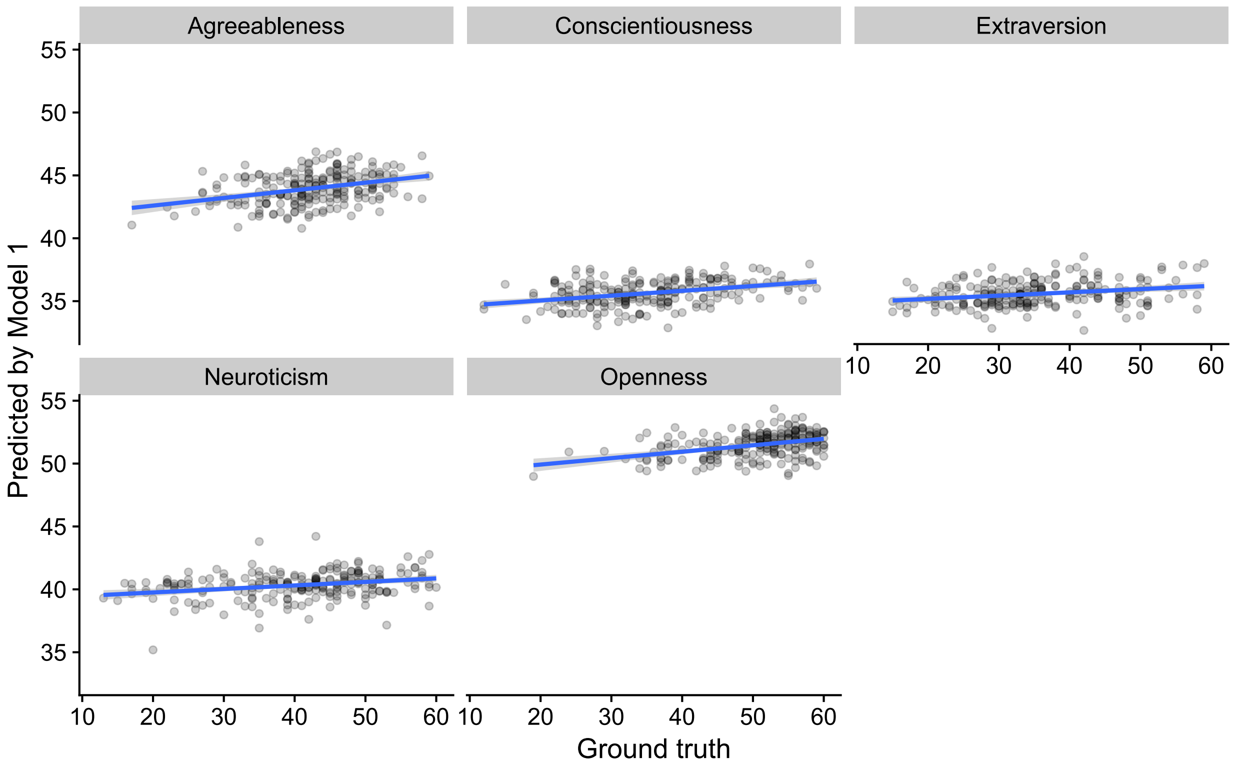

Figure S2

Scatter plot of Model 1's performance (N test set = 215). The X-axis is the ground-truth personality, the Y-axis is the predicted personality by Model 1. The blue line denotes the regression line.

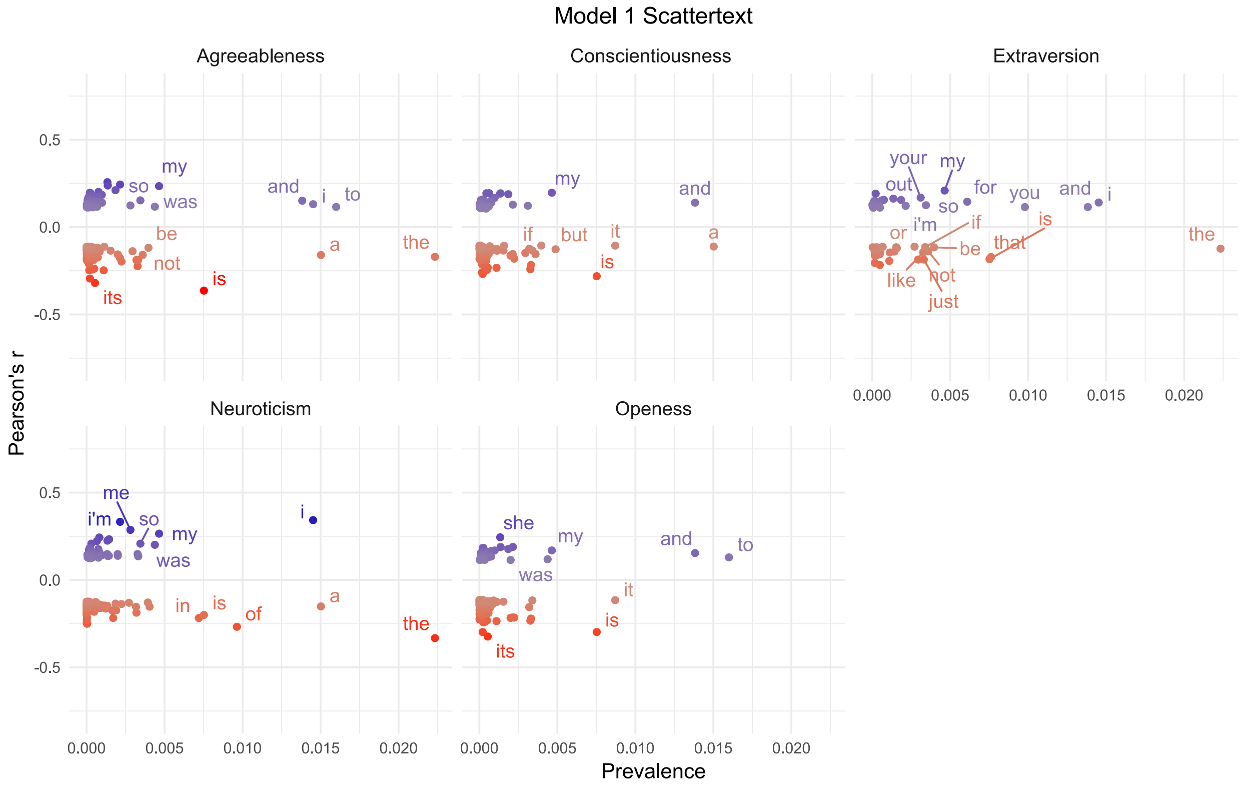

Figure S3

Scattertext of Model 1. The top 1000 features that best predict each personality dimension. The X-axis corresponds to the proportion (prevalence) of the word in the corpus. The Y-axis corresponds to the effect size (value of r), also highlighted by the color wherein blue denotes a positive association and red denotes a negative association.

	Ext	Agr	Con	Neu	Ope
Analytic	−0.07	−0.09	−0.1	0.11	−0.1
Clout	0.06	0.09	0.08	−0.01	0.06
Authentic	0.01	−0.05	−0.05	0.16	−0.07
Tone	−0.11	−0.3	−0.27	0.12	−0.35
WPS	−0.18	−0.27	−0.28	0.2	−0.25
Sixltr	−0.15	−0.31	−0.3	0.21	−0.34
Dic	−0.23	−0.25	−0.24	−0.03	−0.19
function	−0.1	−0.09	−0.07	0.02	0
pronoun	0.1	0.06	0.1	−0.12	0.04
ppron	0.18	0.26	0.24	0.15	0.21
i	−0.11	−0.13	−0.11	−0.07	−0.11
we	0.2	0.2	0.26	−0.19	0.22
you	0.09	0.24	0.17	0.15	0.2
shehe	0.05	0.17	0.17	−0.13	0.19
they	0.08	0.16	0.14	−0.06	0.1
ipron	0.09	0.26	0.21	−0.01	0.24
article	0.07	0.13	0.09	0.09	0.07
prep	−0.06	0.07	−0.03	0.31	0.04
auxverb	0.09	0.26	0.2	0.3	0.26
adverb	−0.05	0.01	−0.01	0.03	0.03
conj	0.02	0.03	0.01	0.05	−0.03
negate	−0.06	0.01	0.02	−0.12	0.09
verb	0.06	0.09	0.09	−0.22	0.07
adj	0.16	0.26	0.23	0.15	0.24
compare	0.1	0.22	0.2	0.02	0.22
interrog	0.1	0.09	0.07	0.13	0.06
number	−0.18	−0.23	−0.25	0.22	−0.18
quant	0.16	0.21	0.17	0.22	0.19
affect	0.11	−0.01	−0.01	0.18	−0.07
posemo	0.14	0.07	0.04	0.29	0.02
negemo	0.14	0.14	0.11	0.16	0.09
anx	0.03	0.04	0.03	0.23	0.05
anger	0.02	−0.08	−0.04	0.06	−0.08
sad	0.12	0.05	0.05	0.22	0.03
social	−0.2	0	−0.09	0.15	0.06
family	0.04	0.2	0.15	0.15	0.24
friend	−0.16	0.01	−0.07	0.1	0.05
female	−0.05	0.09	0.02	0.16	0.08
male	−0.19	−0.09	−0.14	−0.02	−0.01
cogproc	−0.02	0.12	0.09	0.21	0.19
insight	−0.17	0.09	−0.02	0.23	0.16
cause	0	0.09	0.07	0.16	0.09
discrep	0.12	0.31	0.23	0.3	0.3
tentat	0.03	−0.01	0	0.2	−0.03
certain	0.12	0.28	0.21	0.32	0.26
differ	0.12	0.32	0.25	0.33	0.31
percept	0.09	0.13	0.09	0.23	0.11
see	−0.28	−0.32	−0.33	0.11	−0.26
hear	0.13	0.28	0.22	0.21	0.27
feel	−0.16	−0.11	−0.17	0.29	−0.06
bio	0.26	0.26	0.26	0.11	0.18
body	0.14	0.17	0.14	0.24	0.13
health	0	0.12	0.1	−0.14	0.15
sexual	−0.12	−0.25	−0.22	−0.14	−0.29
ingest	−0.08	0.04	0.01	0.19	0.1
drives	−0.12	0	−0.04	−0.09	0.05
affiliation	0.05	0.17	0.14	0.02	0.19
achieve	0.01	0.27	0.14	0.49	0.24
power	−0.18	−0.05	−0.13	0.27	−0.01
reward	−0.02	0.24	0.1	0.23	0.26
risk	−0.04	0.21	0.09	0.32	0.19
focuspast	−0.07	0.17	0.04	0.35	0.17
focuspresent	0.05	0.32	0.26	−0.06	0.39
focusfuture	−0.1	−0.03	0.01	−0.25	0.07
relativ	−0.12	0.08	0	0.27	0.15
motion	−0.13	0.04	−0.02	0.05	0.07
space	0.07	0.26	0.19	0.21	0.25
time	0.14	0.1	0.09	0.12	0.04
work	0.08	0.12	0.14	−0.03	0.12
leisure	0.19	0.32	0.22	0.46	0.24
home	0.21	0.42	0.3	0.49	0.34
money	0.13	0.39	0.26	0.53	0.35
relig	0.01	0.41	0.25	0.41	0.45
death	0.1	0.49	0.34	0.49	0.53
informal	−0.06	−0.26	−0.14	−0.3	−0.15
swear	−0.13	−0.2	−0.19	0.09	−0.19
netspeak	0.31	0.29	0.31	−0.05	0.2
assent	0.11	0.28	0.2	0.23	0.25
nonflu	0.15	0.16	0.19	−0.07	0.14
filler	−0.02	−0.23	−0.09	−0.53	−0.18

Corr: 1.0, 0.5, 0.0, −0.5, −1.0

Figure S4

Model 1 (personality from produced text): Zero-order correlations of predicted personality dimensions with LIWC categories. Blue cells denote negative associations; red cells denote positive associations.

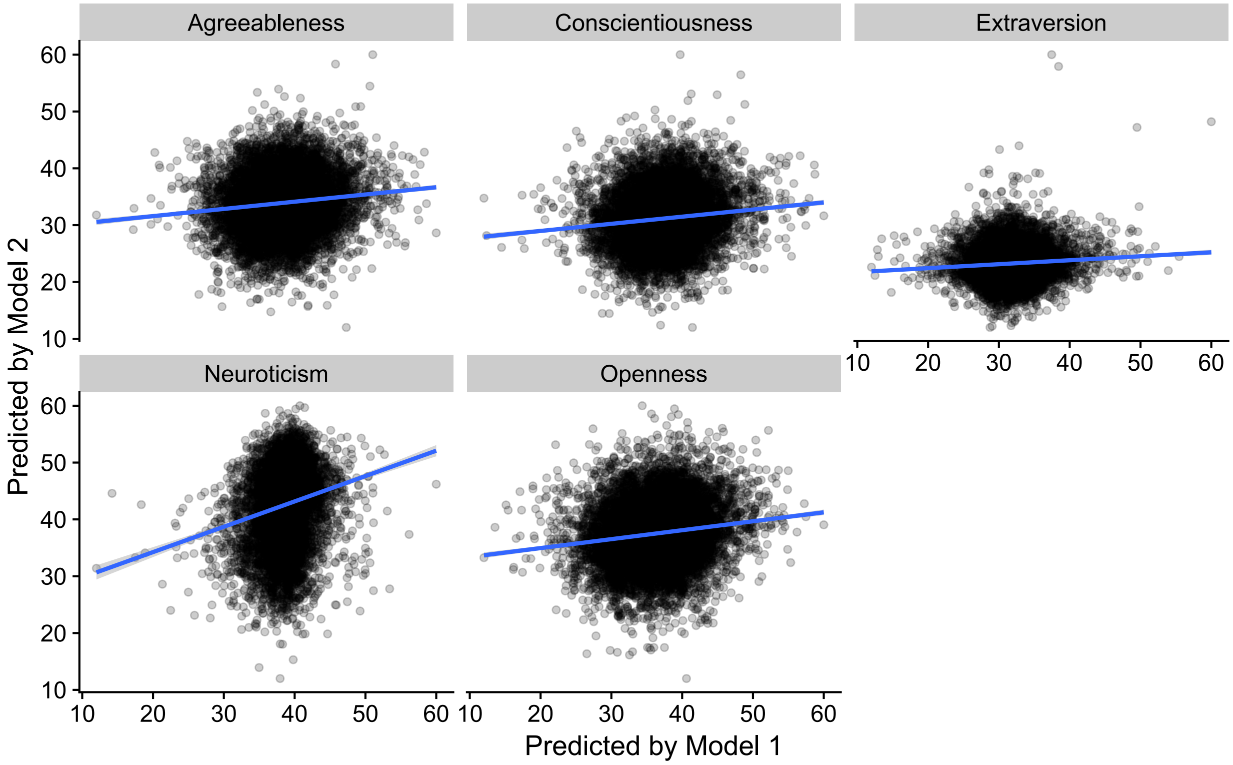

Figure S5

Scatter plot of Model 2's 5-fold cross-validation performance (N = 10,050). The X-axis is the predicted personality by Model 1, the Y-axis is the predicted personality by Model 2. The blue line denotes the regression line.

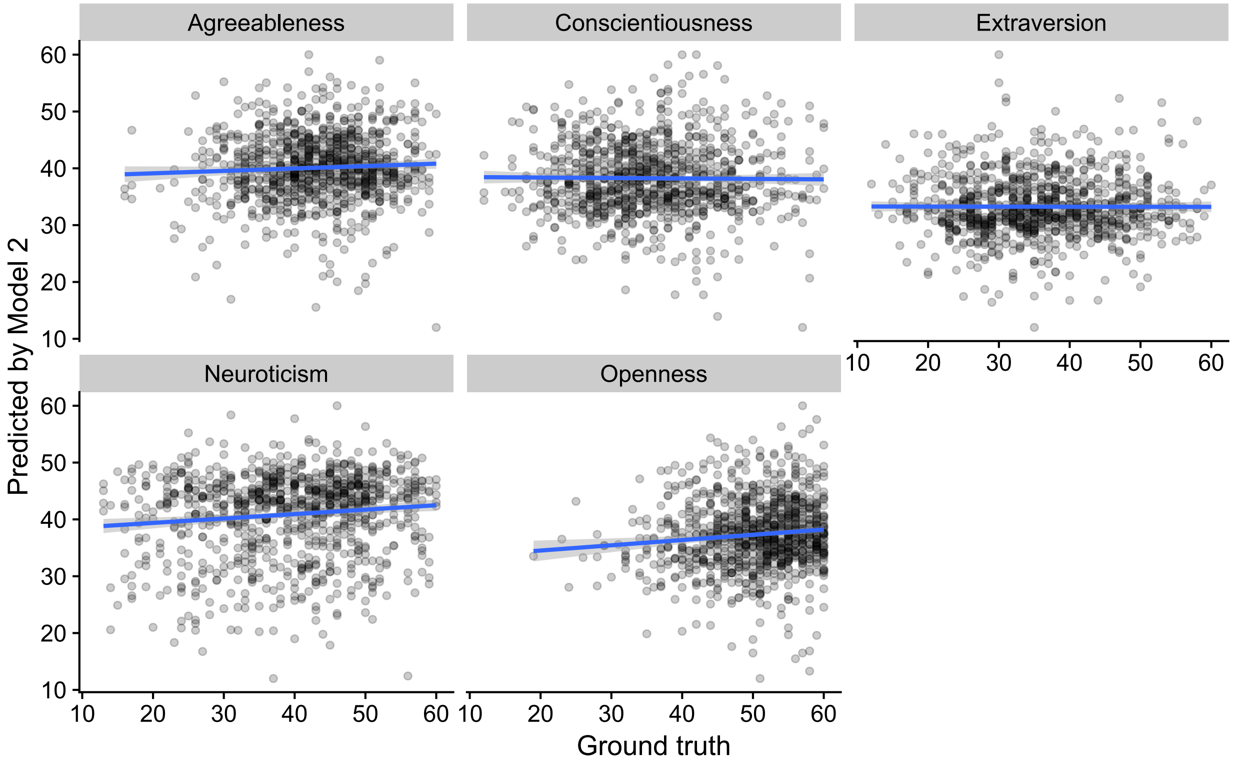

Figure S6

Scatter plot of Model 2's performance against seed data ground-truth (N = 939). The X-axis is the ground-truth personality, the Y-axis is the predicted personality by Model 2. The blue line denotes the regression line.

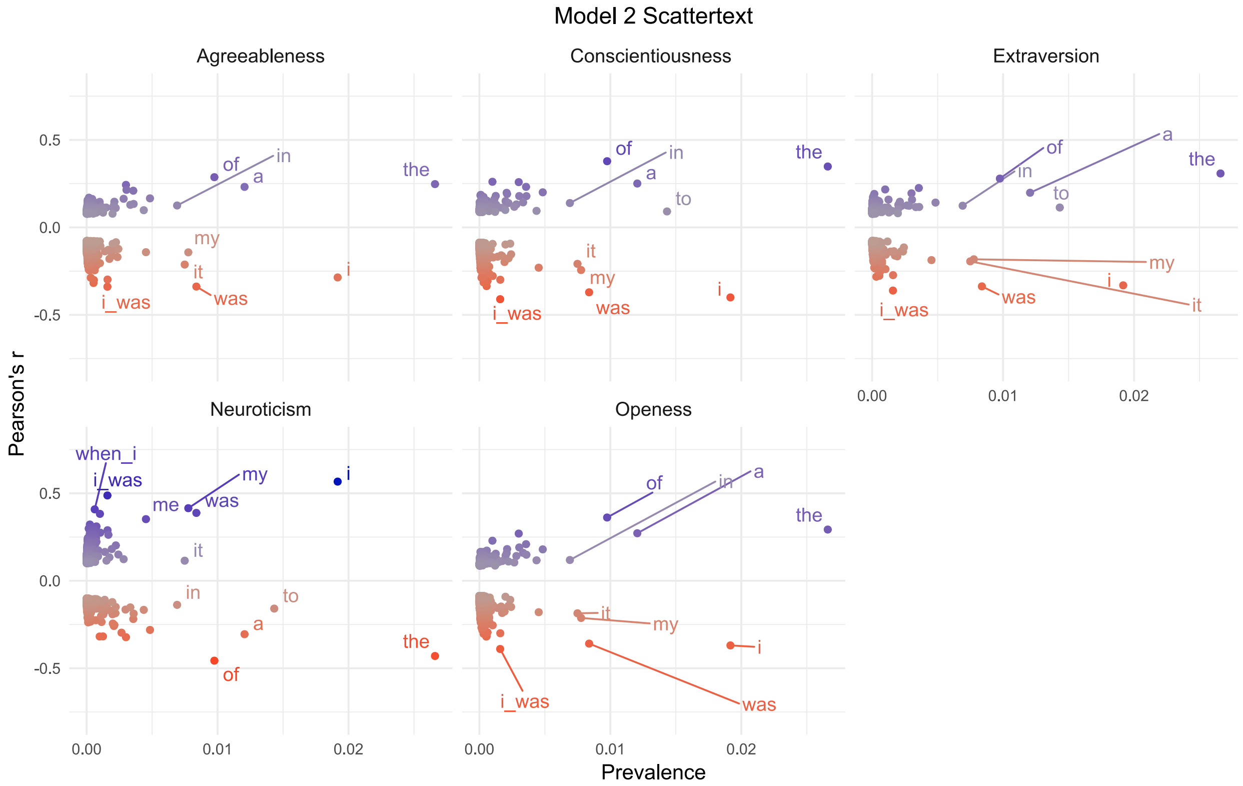

Figure S7

Scattertext of Model 2. The top 1000 features that best predict each personality dimension. The X-axis corresponds to the proportion (prevalence) of the word in the corpus. The Y-axis corresponds to the effect size (value of r), also highlighted by the color wherein blue denotes a positive association and red denotes a negative association.

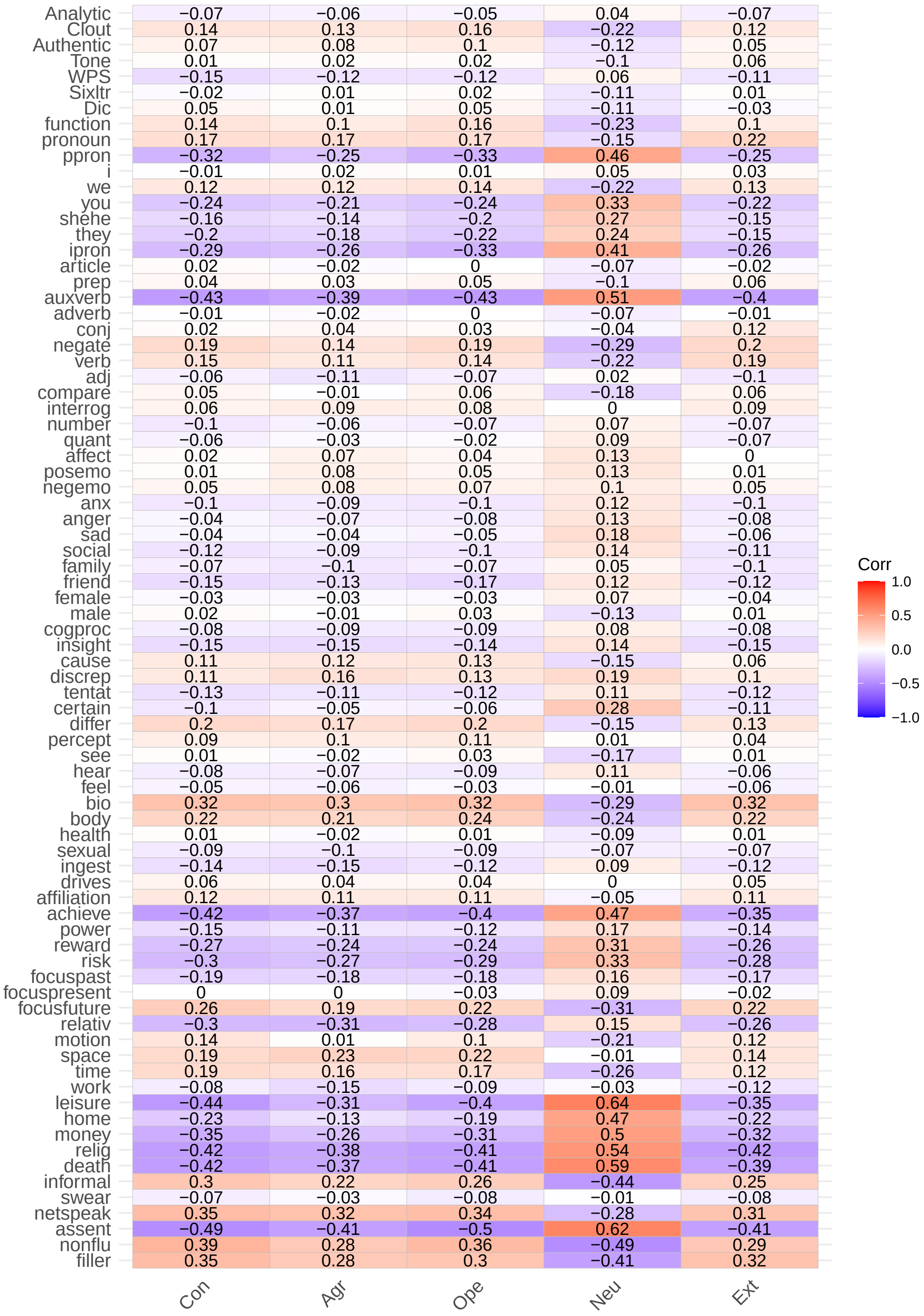

Figure S8

Model 2 (personality from consumed text): Zero-order correlations of predicted personality dimensions with LIWC categories. Blue cells denote negative associations; red cells denote positive associations.

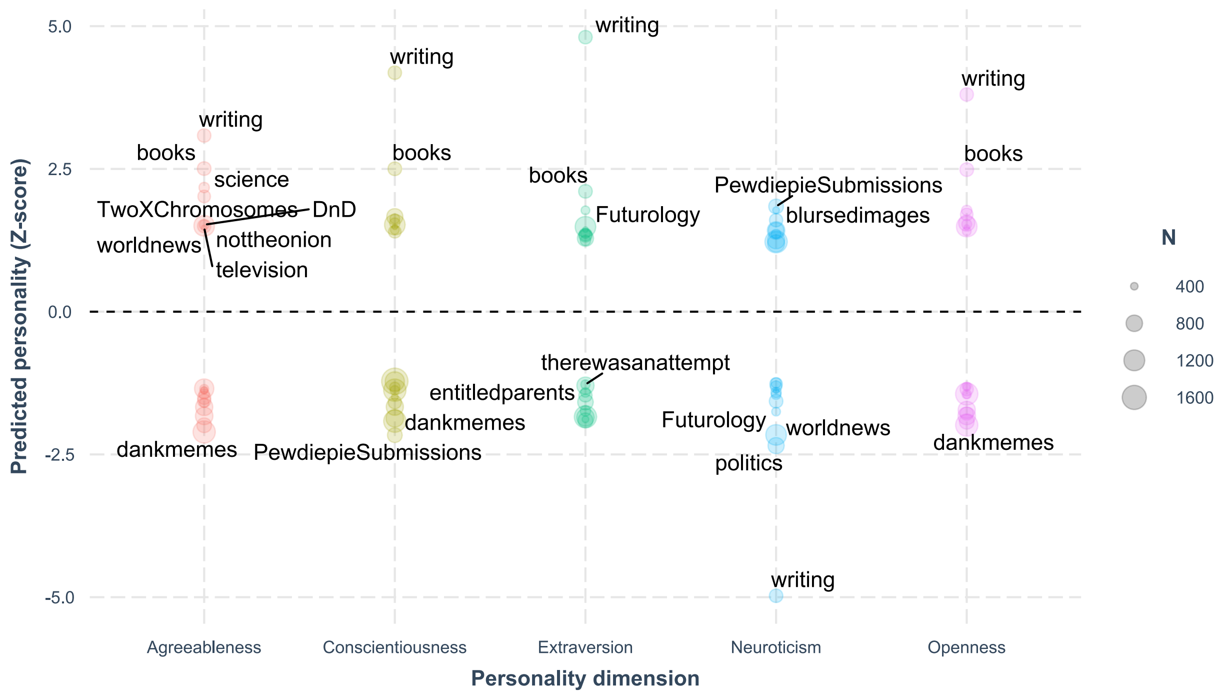

Figure S9

Visualizing the 20 most and least popular subreddits by personality dimension (based on 11,820 users with Model 2 predictions). The scores are the predicted z-score of Model 2's prediction. The dashed line denotes 0. The size of plotting symbol denotes N, the number of contributing users in our sample to each subreddit.

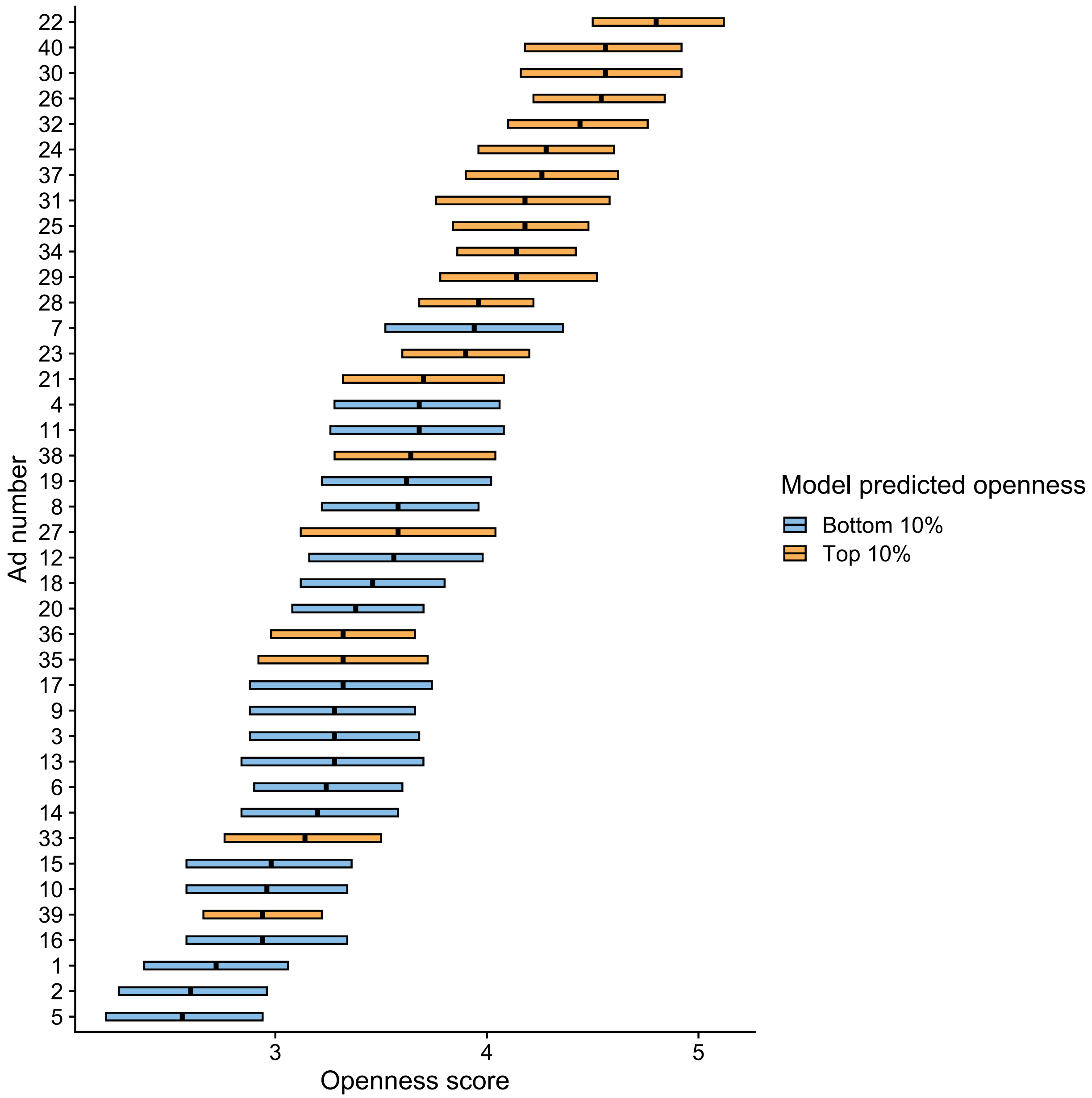

Figure S10

Openness ratings of ads by human raters. The X-axis shows the mean rated openness score and bootstrapped 95% confidence intervals. The Y-axis shows the arbitrary ad number (ads are sorted from highest to lowest rated openness). Color denotes the predicted openness by Model 2: blue for the low openness (bottom decile), orange for high openness (top decile).
